# Bilinguals process incoming words using distributions across both languages

**DOI:** 10.1017/S1366728925100333

**Published:** 2025-07-23

**Authors:** Sarah Frances Phillips, Ailís Cournane

**Affiliations:** 1 Department of Neurology, Georgetown University Medical Center, Washington, DC, USA; 2 Department of Linguistics, New York University, New York, NY, USA

**Keywords:** parsing, language comprehension, code-switching, visual world paradigm, grammatical gender

## Abstract

How does the bilingual experience affect online processing? The distribution of lexical items shared between monolinguals and bilinguals can differ greatly. One critical difference is how code-switching allows more variability in the relative co-occurrence of words. The current study uses a visual world paradigm to test whether the relative distribution between Spanish gender-marked determiners (“el,” “la”) and the non-marked English determiner (“the”) predict the Spanish–English bilingual’s ability to predict and/or integrate an incoming noun. While we replicate a previously observed asymmetry among Spanish–English bilinguals between the masculine “el” and feminine “la,” our cluster permutation test results reveal differences in how bilinguals predict and integrate nouns when preceded by “el” versus “la” or “the.” Comparing our results to existing corpus data, we argue that bilinguals rely on the distributional norms they experience across both single-language and code-switched contexts to facilitate online processing.

## Highlights

- Code-switches between determiners and nouns do not affect online processing
- Spanish–English bilinguals do not use gender marked determiners to predict nouns
- Spanish–English bilinguals use distribution norms of determiners to predict nouns
- Cluster permutations suggest that competition of phonological forms affects processing

## 1. Introduction

Online language processing occurs rapidly and incrementally (Marslen-Wilson, 1975), relying on our ability to integrate what we have perceived and predict what is incoming (Ferreira & Chantavarin, 2018). Most theories emphasize these two mechanisms during processing to account for how effective and efficient knowledgeable language users are during comprehension (Kuperberg & Jaeger, 2015). However, bilinguals seem to defy current models of language processing by how they can readily comprehend (Beatty-Martínez & Dussias, 2017) and produce (Johns & Steuck, 2021) code-switches. Many in the bilingualism literature agree that bilinguals exhibit language non-selectivity (Kroll et al., 2013), that is, they activate words across both of their languages during both production and comprehension. Incorporating language non-selectivity with a working model of online comprehension presents a particular challenge. *How do bilinguals predict and integrate words from both of their languages during processing?* Recent work with Spanish–English bilinguals suggests that the degree of semantic constraint from a sentential context (Lauro & Schwartz, 2017) and the amount of code-switching typically used in one’s environment (Beatty-Martínez & Dussias, 2017; Valdés Kroff et al., 2017) modulate online processing. The current study uses eye-tracking to investigate how lexical knowledge, developed from bilingual experiences, affects one’s ability to predict and integrate an incoming noun based on a preceding determiner.

### 1.1. Prediction and integration for monolinguals and bilinguals

The parser, a component of the language processing mechanism, uses syntactic/semantic information to generate multiple abstract, hierarchical structures in parallel, ranked by the probability of how accurately they match what is intended by the speaker (Traxler, 2014). As linearly perceived input gets integrated into the hierarchical structures under construction, we use the newly updated representations to predict incoming input. Studies measuring eye gaze patterns in a visual world paradigm have shown that manipulating intended representations by varying what word appears at a particular position in the hierarchical structure can affect a person’s ability to predict and/or integrate input (Kamide, 2008).

Altmann and Kamide (1999) presented participants with a visual world with several objects as they heard sentences that manipulated the verb (“the boy will move the cake” versus “the boy will eat the cake”). Because the cake was the only edible object in the visual world, participants shifted their eye gaze more quickly to the cake upon hearing “eat.” Their results demonstrate how the selective nature of verbs used in a restricted context can be informative for prediction. Variations of this paradigm have been used to test for other grammatical features that would affect prediction in particular.

Lew-Williams and Fernald (2007) presented Spanish-speaking toddlers and adults two objects in a visual world as they heard sequences like “*encuentra el zapato*” (=find the_masc_ shoe_masc_). Half of the trials presented two objects that matched for grammatical gender (e.g., *zapato* [=shoe_masc_], *carro* [=car_masc_]), and the other half of the trials presented two objects that differed by grammatical gender (e.g., *zapato* [=shoe_masc_], *galleta* [=cookie_fem_]). Both toddlers and adults shifted their gazes more quickly to the target object during trials when the two objects differed by grammatical gender, suggesting that both groups exploit the informativity of a determiner when used in a restricted context. The authors present three possible explanations for why gendered determiners would facilitate processing in both toddlers and adults. The first account they present is a semantic account, such that gender-marked determiners pre-activate the relevant semantic categories of the subsequent noun; the second account they present is a grammatical account, such that the grammatical rules regarding gender-marking support anticipation of a gendered noun when given a marked determiner; and the third account is a probabilistic account, such that a high probability of co-occurrence between specific words, like determiner-noun pairs, facilitates online processing.

Evidence from other related studies using languages that exhibit grammatical gender agreement more strongly supports the grammatical account (Friederici & Jacobsen, 1999). Incorporating the grammatical account into a model of parsing for bilinguals would require a framework with code-switching-specific constraints for determining how mixed-language constructions are ranked against each other. For example, Parafita Couto et al. (2024) use the Matrix Language Frame Model (Myers-Scotton, 1997, i.a.) and their verbal inflection constraint to account for why bilinguals exhibit gradience in their preferences for switches around a determiner. Their approach would predict switching as being costly, but variably so depending on the syntactic site at which a switch occurs.

Prior work on bilinguals suggests that the syntactic site between determiners and nouns (e.g., *el_SP_* dog_ENG_) may be an especially costly site for switching. Byers-Heinlein et al. (2017) performed a similar task as Lew-Williams and Fernald (2007) but with French–English bilingual toddlers and adults to test for effects of language switching on processing. When grammatical gender is controlled between the two objects presented visually, both toddlers and adults look less at a target object when the noun phrase switched languages (“find the *chien*” [=find the dog_masc_]) compared to when the noun phrase maintained the same language (“find/*trouver* the dog”). However, Spanish–English bilingual toddlers performing a similar task instead exhibit asymmetrical effects between switches that occurred from their dominant versus nondominant language (Potter et al., 2018). Potter and colleagues (2018) argue that the asymmetry between whether the sentential frame was in the dominant or nondominant language suggests that bilinguals exhibit processing differences that reflect how robust their lexical knowledge is. However, the current study tests processing in adults with similar stimuli. We assume that adults have reached a relatively stable, mature state of lexical and grammatical knowledge, compared to children who are still developing that knowledge and often asymmetrically by language. Therefore, on the grammatical account, we expect processing costs to occur more uniformly from one language to the other (Spanish Determiner-English Noun, English Determiner-Spanish Noun) for adults than children (Potter et al., 2018).

### 1.2. Observed similarities/differences between monolinguals and bilinguals

Do bilinguals pattern like monolinguals? Valdés Kroff et al. (2017) used a visual world paradigm to test whether Spanish–English bilingual adults also exhibit prediction effects for grammatical gender. The authors had both Spanish monolinguals and Spanish–English bilinguals complete the same task, where they looked at two objects while listening to variations of “find the object” expressions in Spanish-only (“*encuentra el dulce*” [=find the_masc_ candy_masc_]). They also had the Spanish–English bilinguals listen to comparable Spanish-to-English expressions that consistently switched languages between the gender-marked Spanish determiner and an English noun (“*encuentra el* candy” [=find the_masc_ candy]). Unlike the Spanish-only trials comparing items that differ in gender only, the code-switched trials compared English nouns that are phonological competitors as well as having translational equivalents differ by grammatical gender (e.g., candy [=*dulce* (masc)] versus candle [=*vela* (fem)] with the expectation that gender facilitatory effects would override phonological competition effects (Dahan et al., 2000). While both groups exhibited greater looks to the target for the feminine-marked determiner “*la*” in the Spanish-only different-gender trials, only the Spanish monolingual group exhibited greater looks to the target for the masculine-marked determiner “*el.*” Furthermore, the Spanish–English bilingual group exhibited greater looks to the gender-matched target only for “*la*” (“*encuentra la* candle”), and not “*el*” (“*encuentra el* candy”), in the Spanish-to-English expressions. The authors argue that the processing differences observed between Spanish monolinguals and Spanish–English bilinguals reflect code-switching experience.

Beatty-Martínez and Dussias (2019) further account for the processing differences observed between Spanish’s masculine “*el*” and feminine “*la*” among Spanish–English bilinguals by arguing that distributional asymmetries in their input, likely caused by code-switching, lead to imbalanced representations of grammatical gender. Spanish–English bilingual communities vary in their gender-assignment strategies for mixed noun phrases (Bellamy & Parafita Couto, 2022). Beatty-Martínez and Dussias (2019) assume that we are all sensitive to distributional information in language (Clayards et al., 2008), such that distributional patterns in production shape comprehension (MacDonald, 2013). From these assumptions, they predict that the degree of variability in the distributions of “*el*” and “*la*” gives rise to an asymmetry in how grammatical gender is represented and, in turn, used during online processing. This differs from assuming that, like Spanish monolinguals, bilinguals have more balanced representations (i.e., the quality of propensity to predict input) of masculine and feminine grammatical genders.

In the Bangor Miami corpus of Spanish–English bilingual speech (http://bangortalk.org.uk/speakers.php?c=miami), there are roughly 8400 instances of articles (“*el*,” “*la*,” “the”) used across all speakers represented. “*El*” and “*la*” are used somewhat equally often (18% and 21% of tokens, respectively), but “the” is used the most frequently (61% of tokens). Parafita Couto and Gullberg (2019) analyzed 2981 noun phrases produced by a subset of bilingual speakers from this corpus, finding ≈45% are Spanish only (*n* = 1354) and ≈3% are mixed Spanish–English noun phrases (*n* = 98). While code-switched noun phrases are generally infrequent, examining the mixed Spanish–English noun phrases produced in the corpus (*n* = 322) reveals a pronounced imbalance: 92% of the mixed noun phrases used “*el*” with an English noun (*n* = 297), 5% used “the” with a Spanish noun (*n* = 16) and 3% used “*la*” with an English noun (*n* = 9) (Valdés Kroff, 2012). Similar imbalances, though not always so heavily asymmetrical across Spanish–English bilingual communities, have been observed in other corpora (Jake et al., 2002; Pfaff, 1979). Switching within a noun phrase occurs infrequently, but “*el*” is most often used in the mixed-language context. A probabilistic account would predict that “*el*” specifically incurs a processing cost because the distributional nature of “*el*” co-occurring with English nouns would prevent masculine grammatical gender from being reliable for prediction.

Despite Valdés Kroff et al. (2017) and Potter et al. (2018) reporting results from two different bilingual populations, we note that the switch location in both of these studies occurred between the selective word (determiner) and the target word (noun). However, we adopt Beatty-Martínez and Dussias (2019)‘s assumptions and present an alternative account focused on the syntactic frame (noun phrase). If we assume the representations of the lexical items used by Byers-Heinlein et al. (2017) and Potter et al. (2018) are well-formed from distributional information in the input (Jusczyk & Aslin, 1995), one could also argue that switching languages is generally dispreferred by the parser (regardless of where the switch occurs syntactically), ranking mixed-language sequences lower due to their relative infrequency. They would therefore incur a processing cost during integration.

### 1.3. Overview of the present study

To answer the larger question of how bilinguals can readily predict and integrate input across their languages, we test whether lexical items (“*el*,” “*la*,” “the”) that belong to a single category (Determiner [det]) elicit differences in the Spanish–English bilingual’s ability to predict and/or integrate an incoming noun.

Our first hypothesis treats language switching as disruptive for the parser, given how infrequently these switches occur. This predicts that code-switched expressions incur processing costs when compared to single-language expressions, regardless of where those switches occur (before the noun phrase, within the noun phrase). Our second hypothesis treats grammatical gender as an informative feature of Spanish–English bilinguals’ knowledge. If adults rely on grammatical knowledge (gender features) to anticipate input, one expects clear differences for the Spanish determiners (which have gender features) compared to the English determiner. This predicts that “*el*” and “*la*” facilitate processing when compared to “the.” Our third hypothesis is developed from a widely accepted idea about our sensitivity to distributions in language. While “*la*” typically co-occurs only with Spanish feminine nouns and “the” typically co-occurs with English nouns, “*el*” commonly co-occurs with both Spanish masculine and English nouns. If Spanish–English bilinguals demote the grammatical gender informativity of “*el*” because its distribution is more variable, one predicts a similar asymmetry between “*el*” and “*la*” as has been observed by Valdés Kroff et al. (2017), even when trials with “the” are interspersed as we do here.

As part of a larger protocol investigating processing differences of code-switched expressions between toddlers and adults, we presented four animals in a visual world paradigm to Spanish–English bilingual adults as they heard variations of “find the animal” expressions. We varied the three-word sequences to create four switching conditions (No Switch [NO], Switch after Word 1 [W1], Switch after Word 2 [W2], Double Switch [2X]) to maintain a 50/50 probability for switching after each word across trials. Doing so allowed us to test whether identifying a target object would be affected by the lexical knowledge of the determiners (grammatical gender, distributions) and/or its combinatory context (switch, no-switch). We performed two sets of statistical analyses. First, we performed a series of repeated measures ANOVAs to determine if we observe effects of switching, grammatical gender, and/or distributional sensitivity. This allowed us to compare our results to prior work that also measured overall differences in proportions of looks to a target. Second, we performed a series of pairwise cluster permutation tests to delineate potential differences between determiners during prediction (earlier) and integration (later) stages of processing. This also allowed us to identify specific time windows where eye gaze behaviors diverged, as compared to prior work that compared performance using binned time windows.

Results from both sets of analyses support our third hypothesis (distributional sensitivity) but not our first (switch cost) or second (grammatical gender) hypotheses. When examining looks to target averaged across the entire trial window, participants exhibited the most looks to target for “*la*” and the fewest looks to target for “*el.*” Contexts where “*el*” was followed by an English noun exhibited fewer looks than when “*el*” was followed by a Spanish noun. Performing the cluster permutation tests revealed a complex pattern for “*el*” that suggests bilinguals take statistics of the distributional cues of words across both single- and mixed-language contexts that can become useful at different stages of online processing.

## 2. Methods

This study is part of a larger protocol investigating bilingual processing in both Spanish–English bilingual adults and toddlers. As we intend to extend this work with 2-year-olds, we designed our stimuli to be maximally engaging and productive for toddlers in both Spanish and English. Here, we focus only on the data collected from bilingual adults.

### 2.1. Participants

Fifty-three Spanish–English bilingual adults from the greater New York City (NYC) area participated in our study. Three participants’ data were excluded due to having less than 75% of their eye gaze data remaining after preprocessing. The self-reported and eye-tracking data that follows come from our remaining 50 participants (*n* = 50: 15 Male, 34 Female, 1 Nonbinary). All participants reported having normal or corrected-to-normal vision as well as native(−like) proficiency in both Spanish and English for inclusion. Descriptive statistics collected using the LEAP-Q (Marian et al., 2007) are provided in Table 1.

**Table 1.** Descriptive statistics of participant sample

Characteristic	M	SD
Age	24 years	7 years
Age of acquisition – English	3.55 years	4.08 years
Age of acquisition – Spanish	1.41 years	2.72 years
Current proportion of exposure – English	.65	.20
Current proportion of exposure – Spanish	.31	.18
Self-rated understanding – English	9.77/10	0.77
Self-rated understanding – Spanish	9.16/10	1.26

*Note: A subset of relevant self-reported information is provided; participants reported exposure in average percentages that should add up to 100% across their languages and proficiency in understanding each language on a scale from 0 (=not proficient at all) to 10 (=highly proficient).*

Most of our participants reported acquiring Spanish first from birth (treated as 0 years) but were English dominant at the time of the study. They varied widely in their Hispanic/Latine identities (e.g., “Colombian,” “Mexican”), with half of our participants more strongly identifying with their Hispanic/Latine identity than their American identity. When asked to report a percentage of the current exposure, on average, for each language, our participants generally reported greater exposure to English than Spanish in their daily lives at the time of the test in NYC. Fourteen of our 50 participants reported acquiring a third language (mostly French or Portuguese), but their reported percentages of exposure to a third language averaged less than 10%. When asked to report their proficiency in understanding each language using a Likert scale from 0 to 10 (where 10 represents the highest proficiency), our participants reported comparably high proficiency in their ability to understand both spoken English and spoken Spanish. Overall, our participants generally represent early, highly proficient bilinguals from various Spanish-speaking communities in the greater NYC area and report greater daily exposure to English (*M* = 65%) than Spanish (*M* = 31%).

### 2.2. Stimuli & experimental design

Participants were presented with both an auditory stimulus and a visual stimulus simultaneously during each trial. Both sets of stimuli are available via OSF (https://osf.io/9wg3b/). Auditory stimuli were three-word sequences that followed the structure “find the animal” (e.g., “find the dog,” “*encuentra el perro*”) and described one of the four animals depicted visually. The four animals were selected (chicken, dog, horse, turtle) based on the productivity of their English labels (e.g., “dog”) and Spanish labels (e.g., “*perro*”) among 2-year-olds as reported in WordBank (Frank et al., 2016), syllable length (English: 1–2 syllables; Spanish: 2–3 syllables) and balanced for their inherent grammatical gender in Spanish (Masculine [masc]: dog, horse; Feminine [fem]: chicken, turtle). The list of animal-denoting nouns selected, accompanied by the proportion of children at 24 months who produce each lexical item, is provided in Table 2.

**Table 2.** Proportion of 2-year-old children producing selected lexical items from WordBank. N.B

English nouns		Spanish nouns	
chicken	.66	*gallina*	.56
dog	.93	*perro*	.87
horse	.79	*caballo*	.79
turtle	.66	*tortuga*	.51

*Note*: The proportion of 2-year-old monolingual English-speaking children producing each English noun and monolingual Spanish-speaking children producing each Spanish noun; we note that the English word “chicken” would normally be translated as “pollo” in Spanish, and “gallina” in Spanish would normally be translated as “hen” in English. We did not use “hen” as it was not as productive for 2-year-old American English––speaking children (Stanford Word Bank (hen[0.15] < chicken[0.65])).

“Find the animal” sequences varied by language for each item to create four different switching conditions: No Switch (NO), Switch after Word 1 (W1), Switch after Word 2 (W2) and Double Switch (2X). This was done to ensure a 50% probability for switching after each word in a given sequence across trials. While “the” was the only English determiner used (50% of all trials), the Spanish determiner varied by the grammatical gender of the Spanish(−equivalent) noun (25% of all trials used “*el*,” and 25% of all trials used “*la*”). Doing so maintained grammatical gender congruence even when switches occurred between a Spanish determiner and an English noun. The switching conditions, balanced for language of items, are exemplified in Table 3. Eight unique sequences were generated from our switching and language conditions for each of our four animals, 32 total sequences across conditions.

**Table 3.** Experimental conditions with example stimuli

Condition	Language (target animal)	Example stimulus		
No switch (NO)	English	find	the	dog
	Spanish	*encuentra*	*el*	*perro*
Word 1 switch (W1)	English	*encuentra*	the	dog
	Spanish	find	*el*	*perro*
Word 2 switch (W2)	English	*encuentra*	*el*	dog
	Spanish	find	the	*perro*
Double switch (2X)	English	find	*el*	dog
	Spanish	*encuentra*	the	*perro*

All auditory stimulus items (including both Spanish and English) were produced by a Spanish–English bilingual consultant who self-reported relatively balanced amounts of exposure to both Spanish and English in her daily life. The consultant speaks a variety of South American Spanish and a variety of North American English. Recordings were made continuously with several repetitions of each lexical item, but single instances of each word were extracted from the recordings using Audacity (www.audacityteam.org/) and then presented separately at time-locked intervals through our SR experiment builder.^1^ The average duration of determiners across both languages is approximately 190 ms, and the average duration of nouns across both languages is approximately 600 ms. We used the longest item for each unit in our “find the animal” sequence to determine the onset timings across trials (W1[“find”]: 800 ms, W2[det]: 400 ms, W3[“animal”]: 800 ms). To give a sense of what these sequences would sound like in a given trial, example concatenations are provided via OSF (https://osf.io/9wg3b/).

**Figure 1.**
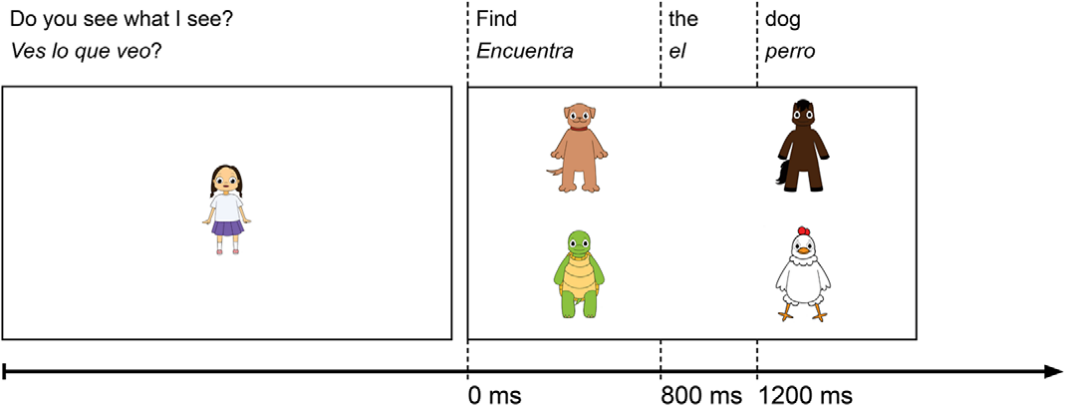
Experimental trial structure with example visual world.

Visual worlds were 8000 × 4500 images at 300 dpi resolution. Each visual world contained four quadrants: top left, top right, bottom right and bottom left. In each quadrant, an image of one of our four animals (chicken, dog, horse, turtle) was placed. Four unique visual worlds were created by shuffling where each animal appeared in one of the four quadrants to avoid gender-matched targets from appearing in the same relative position to each other. All images of our animals were anthropomorphized into a standing position to maintain similar body positions and the relative location of their eyes was consistent. An example of a visual stimulus item as presented during the trial sequence is provided in Figure 1. Visual stimuli were created by a digital illustrator consultant using Procreate (procreate.art/) on an iPad Pro.

### 2.3. Procedure

After obtaining informed consent, all participants were seated approximately 18–24 inches away from an EyeLink Portable Duo eye-tracker. The tracker sat just below a 24-inch, 1080-*p* BenQ monitor, with the top edge of the eye tracker just below the bottom edge of the monitor. Before starting the experiment, participants were asked to place a target sticker on their forehead and lower the top edge of facial masks (required as part of our COVID-19 safety protocol) to just cover the tips of their noses.

Each session began with calibration using five fixation points that were subsequently validated. At the beginning of each experiment, participants met a narrator, Sylvia, who introduced the four target animals in both English and Spanish and gave instructions for the task. This was done to ensure participants were familiar with the intended English and Spanish labels (e.g., “chicken” and “*gallina*”) for each animal (e.g., image of chicken). Before each trial, the experimenter performs drift correction. Each trial starts with Sylvia as a fixation point in the center of the screen, followed by a visual world with four objects: a target object (e.g., dog), a gender distractor object (e.g., horse) and two filler objects (e.g., turtle and chicken). During the presentation of a visual stimulus item, the participant hears an auditory stimulus item describing the target object. Participants were asked to locate the target object using their eyes. After participants completed the eye-tracking session, they filled out the LEAP-Q (Marian et al., 2007). Participants typically spent 30 minutes in the lab and were paid $10 for their participation.

### 2.4. Data acquisition

All experimental sessions recorded only the left eye of each participant using remote tracking. Eye data, including gaze position, saccades and fixation events, were sampled at 1000 Hz. Each participant’s eye-tracking information was acquired on a host computer (IBM Thinkpad), forwarded to a display computer (Mac Mini) through an Ethernet cable and compiled into an EDF file. The experiment was designed and presented using SR Research Experiment Builder (“SR Research Experiment Builder 2.2.1”, 2019).

### 2.5. Data preprocessing & analyses

All EDF files were first preprocessed using the EyeLink Data Viewer by generating a sample output report. This report includes the following data for each sample collected at 1000 Hz: time, eye gaze location in (*x*,*y*) coordinates, velocity, acceleration and pupil size. Fixations and saccades were also segmented in Data Viewer using EyeLink’s standard algorithm using velocity (30°s^−1^) and acceleration (8000°s^−1^) thresholds. All data points were saved into a new TXT file for each participant. Further preprocessing, analyses and visualizations were performed in R and RStudio (v.1.3.1093) using the VWPre (Porretta et al., 2017) and eyetrackingr (*eyetrackingr.com/*) packages. Individual participant data (stored as TXT files) were compiled into one large data frame. Trial data were aligned to the onset of the first word presented in each trial, find. We then removed trials with more than 25% of eye gaze information missing (3.26% removed) and down-sampled our data from 1000 Hz to 5o Hz (generating 20 ms bins).

Our statistical analyses were designed to test two hypotheses: whether switching languages is costly (NO > W1, W2 > 2X) and whether grammatical gender is informative (“el” & “la” > “the”) during prediction and/or integration. We then compare our results to the existing corpora as described in the introduction to infer whether any observed processing differences reflect the distributional norms of our determiners (“*la*” > “*el*” & “the”).

To test whether switching languages is generally costly, we performed a two-way, repeated measures ANOVA over the logit-adjusted values representing proportion of looks to the target animal from the onset of “find” to the end of the trial (0–3500 ms).^2^ We treated switch location (NO, W1, W2, 2X) and determiner (“the,” “*el*,” “*la*”) as factors. The relevant prior work has examined language switch effects on looks to target from 360 ms after the onset of the target noun (Byers-Heinlein et al., 2017; Potter et al., 2018) and effects of determiner on looks to target in binned windows from the offset of the determiner (Valdés Kroff et al., 2017). Doing so implies that any switch costs are related to switches between a determiner and its subsequent noun. However, we added a switch manipulation between “find” and the determiner. If switching is generally costly, there may be switch effects incurred outside of the time window of interest (during/after presentation of the noun). Thus, we include the presentation of “find” in our original ANOVA analysis. We also conducted an ANOVA over the time window of interest from 200 ms after the onset of the determiner through the presentation of the noun (1000–2500 ms) to test whether switch effects are task specific (i.e., locating the target animal) or graded due to having both switch directions (Spanish–English, English–Spanish) incorporated (Parafita Couto et al., 2024). If differences emerge during either prediction or integration for language switching^3^ and/or for determiner (either due to gender informativity or distributional norms), we would expect differences in average looks to the target during our analysis window. However, averaging looks over the entire analysis window would not reveal differences during prediction versus integration of the noun specifically.

To test whether grammatical gender is informative during prediction and/or integration, we performed a series of cluster permutation tests (Maris & Oostenveld, 2007) to identify time clusters where looks to target significantly diverged between each pair of determiners being compared (“the” < “el,” “the” < “la,” “el” = “la”) as well as whether the noun following each determiner was in Spanish or English. We calculated a t-threshold using a Bonferroni-corrected *p*-value for multiple comparisons, and then performed paired t-tests on each 20 ms bin across our analysis window to identify clusters where the difference in means in proportion of looks to the target had a *t*-value above our threshold (*t* > 2.01). Those clusters were then compared to a null distribution generated from 1000 permutations of our data. We report p-values for clusters appearing after the onset of the noun (1200 ms) that reflect the proportion of permuted clusters with larger differences in means than our actual cluster(s).

There is no definitive time course for when prediction and integration processes occur during online comprehension. Recent studies using electrophysiological measures (which also have high temporal resolution) seem to reflect earlier effects of predicting phonological forms and later effects of integrating words into a sentential context (Brodbeck et al., 2022; Gwilliams et al., 2023; Mantegna et al., 2019). Based on their results, we assume here that prediction of the noun would affect proportion of looks to target during the presentation of the target noun (between 1200 and about 1800 ms), and integration of the noun would affect proportion of looks to target after the offset of the target noun’s presentation (about 1800 ms). If grammatical gender facilitates prediction, for example, “*el*” and “*la*” should elicit greater looks to the target between 1200 and 1800 ms when compared to “the.” If the choice of determiner affects ease of integration for Spanish nouns versus English nouns, we expect greater looks to target between 1800 and 3500 ms. What is exploratory is whether distributional norms of our determiners are informative during prediction (1200–1800 ms) and/or integration (1800–3500 ms).

## 3. Results

**Figure 2.**
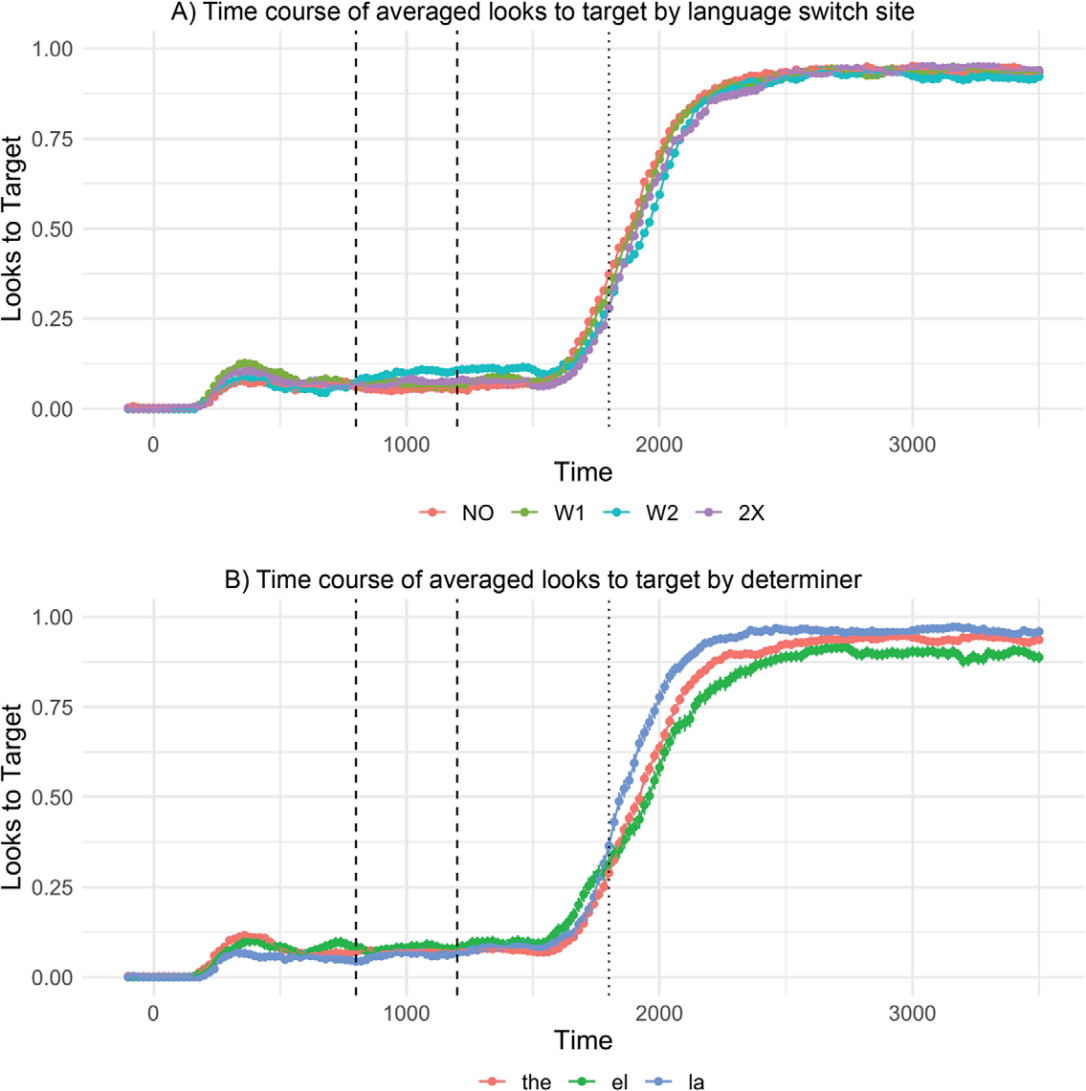
Proportion of looks to target by (A) language switch and by (B) determiner - 20-ms binned proportion of looks to the target object was averaged across participants by each condition used in the auditory stimuli. The dashed lines indicate onset of determiner at 800 ms and onset of noun at 1200 ms. The dotted lines indicate the approximate offset of the noun at 1800 ms. Results from our two-way, repeated measures ANOVA revealed a main effect of determiner and not switch site.

We performed a two-way, repeated measures ANOVA over our entire window of analysis (0–3500 ms) to determine if switching is generally costly (predicting a main effect of switch site) or if switch costs are specific to our region of interest (predicting an interaction effect between switch site and determiner). The averaged looks to target across participants for each 20-ms bin between 0 and 3500 ms by switch site (A) and by determiner (B) are plotted in Figure 2. There was no main effect of switch location on participants’ looks to the target (*F*(3,147) = 1.95, *p* = .12, *η*2 = .04). This result suggests that language switching does not modulate online processing generally.

**Figure 3.**
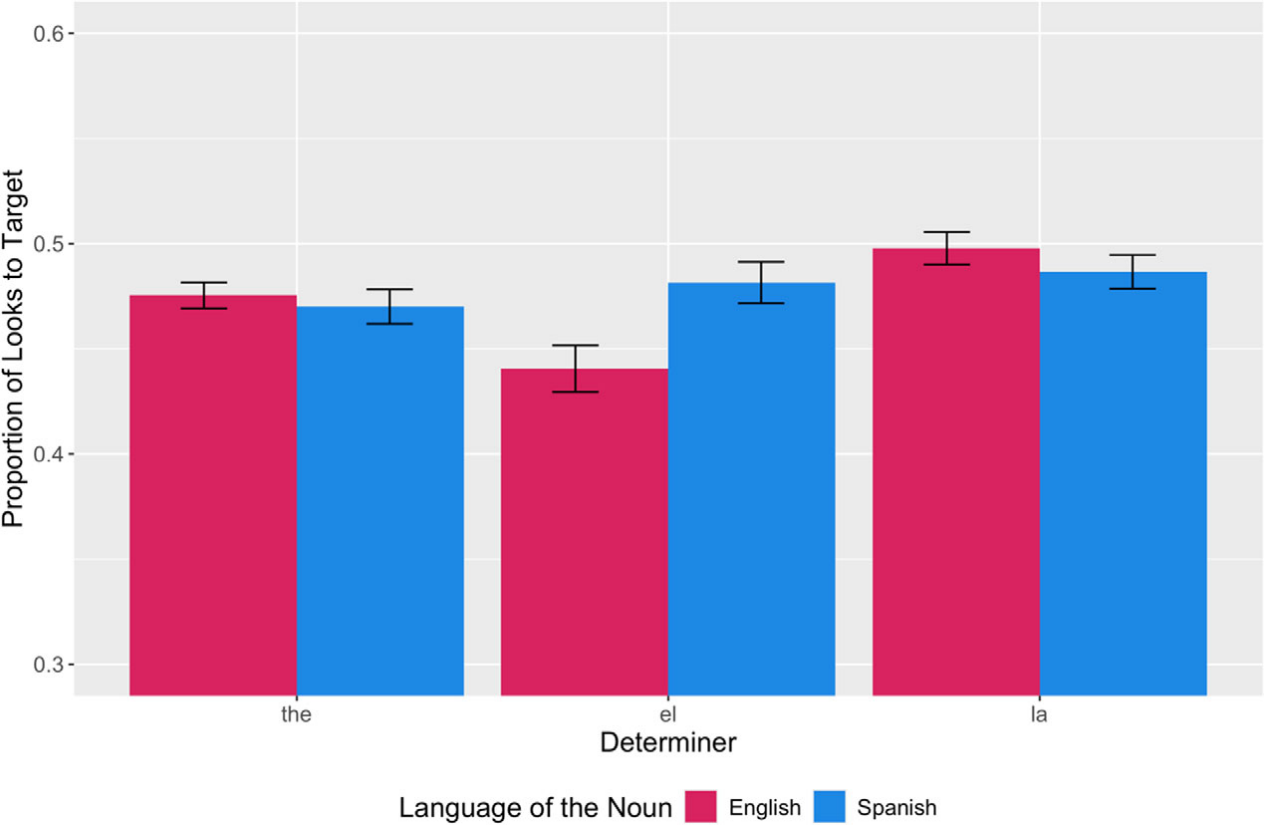
Averaged proportion of looks to target by determiner and language of the noun - Participants looked at the target least for “el” and most for “la.” Unlike “the” or “la,” and participants looked less at the target for “el” when the following noun switched to English (e.g., “encuentra el dog”) and more when the following noun stayed in Spanish (e.g., “encuentra el perro”).

However, there was a significant main effect of determiner on participants’ looks to target (*F*(2,98) = 7.46, *p* = .001, *η*2 = .13). A bar plot of averaged looks to target by determiner and language of the noun is presented in Figure 3. Estimated marginal means (EMMs) were calculated to perform pairwise comparisons between determiners. Pairwise comparisons revealed a significant difference between “*el*” and “*la*” (*p* = .001) and a trending difference between “the” and “*la*” (*p* = .06). These results suggest that participants generally look more to the target upon hearing “*la*” and less upon hearing “*el.*”

There was also a significant interaction between switch location and determiner (*F*(6,294) = 3.20, *p* = .005, *η*2 = .06). EMMs were calculated to perform pairwise comparisons between determiners with respect to each switch condition. Pairwise comparisons revealed significant differences for “*el*” between the no switch (“*encuentra el perro*”) and W2 switch (“*encuentra el* dog”) conditions (*p* = .002); the no switch and 2X switch (“find *el* dog”) conditions (*p* = .007); the W1 switch (“find *el perro*”) and W2 switch conditions (*p* = .004); and the W1 switch and 2X switch conditions (*p* = .02). There were no significant differences between switch conditions for each of the other determiners (“*la*,” “the”). These results suggest that participants generally look more to the target when “*el*” was followed by a Spanish noun (“*perro*”) than an English noun (“dog”).

To further test whether switch costs are specific to the noun phrase, we conducted the same ANOVA post-hoc but for the target time window of interest, the offset of the determiner through the presentation of the noun (1000–2500 ms), instead of the entire trial window. Results revealed a main effect for switching (*F*(3,147) = 2.71, *p* = .05, *η*2 = .05), a main effect for determiner (*F*(2,98) = 13.78, *p* < .001, *η*2 = .22) and a significant interaction between our two factors (*F*(6, 294) = 4.80, *p* < .001, *η*2 = .09). While these results suggest that looking behaviors were affected by switching, it seems that differences were mostly due to the determiner used, given the large effect size (*η*2 > .14) of the determiner, further modulated by the effects of switch, given the medium effect size (*η*2 > .06) of the interaction.

EMMs were again calculated to perform pairwise comparisons between determiners and the switch site for our target time window of interest. Pairwise comparisons again revealed significant differences for “*el*” the no switch (“*encuentra el perro*”) and W2 switch (“*encuentra el* dog”) conditions (*p* < .001); the no switch and 2X switch (“find *el* dog”) conditions (*p* < .001); the W1 switch (“find *el perro*”) and W2 switch conditions (*p* = .008); and the W1 switch and 2X switch conditions (*p* = .002). Again, there were no significant differences between switch conditions for each of the other determiners (“*la*,” “the”). These results align with our original set of results conducted on the entire trial window, suggesting that participants generally look more to the target when “*el*” was followed by a Spanish noun (“*perro*”) than an English noun (“dog”).

**Figure 4.**
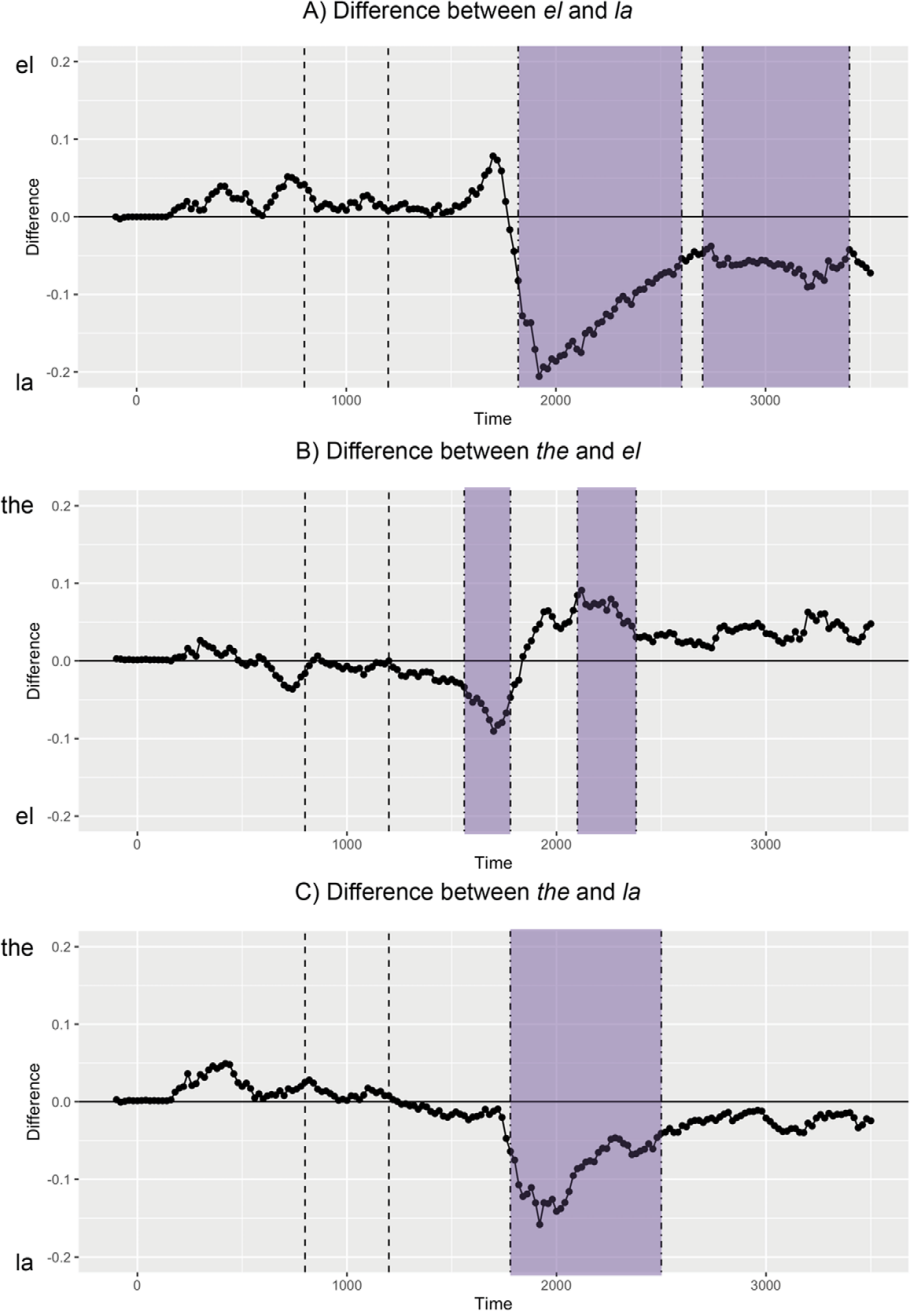
Pairwise time cluster permutation results by determiner - Significant time clusters from permutation tests between “el” and “la” (A), “the” and “el” (B), “the” and “la” (C) are highlighted in purple.

**Figure 5.**
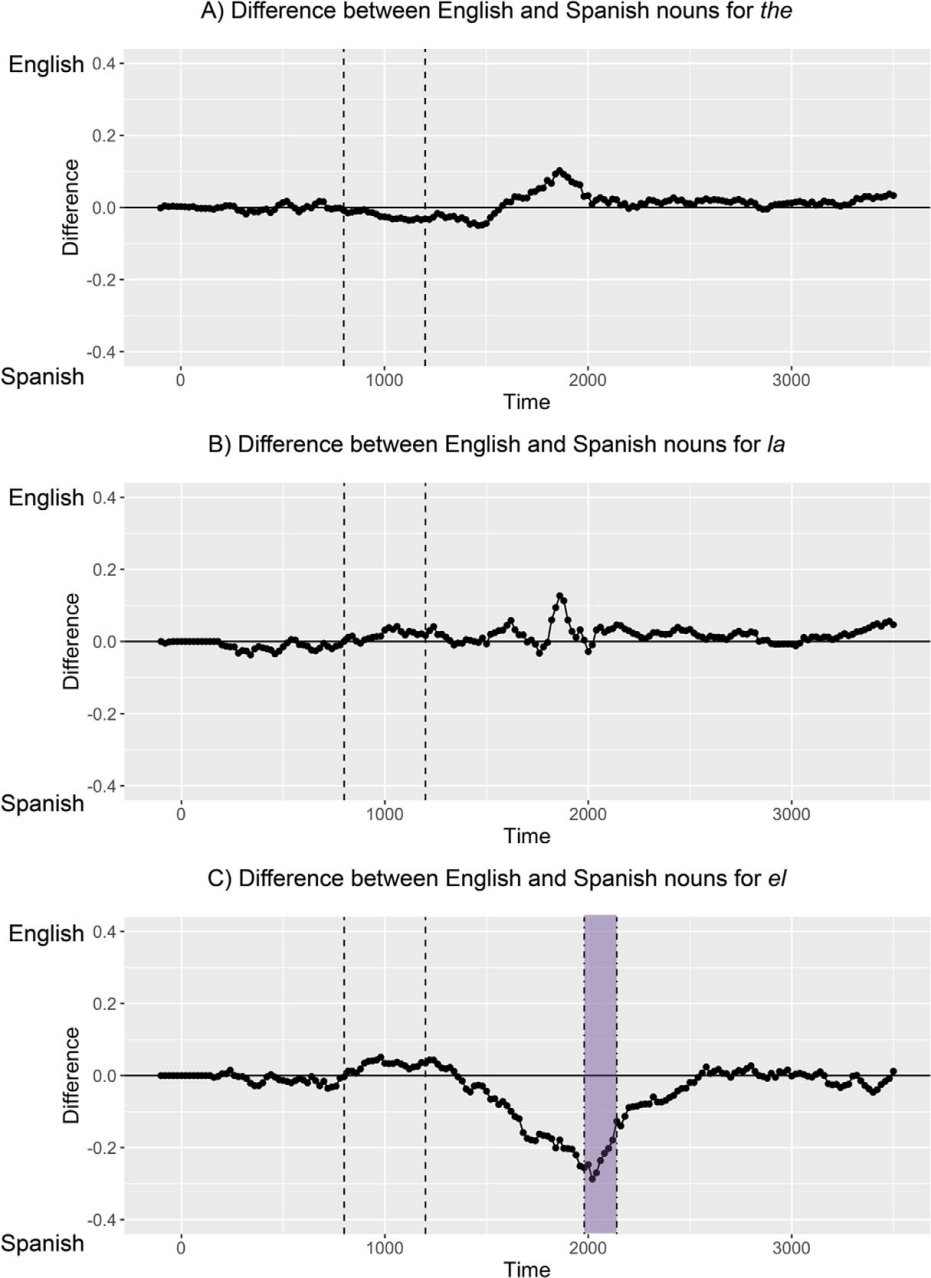
Pairwise time cluster permutation results by language of the noun for each determiner - No significant time clusters emerged from permutation tests between English and Spanish nouns for “the” (A) or “la” (B), but one significant time cluster emerged for “el” as highlighted in purple (C).

We performed a series of cluster permutation tests to determine whether differences elicited by our determiners occurred during prediction or integration. Differences in looks to target by each determiner pair are depicted in Figure 4; and differences in looks to target by noun (English, Spanish) for each determiner are depicted in Figure 5.

When we compared “*el*” and “*la*,” two significant time clusters emerged. Participants looked more at the target for “*la*” than “*el*” between 1820 and 2600 ms (*p* < .001) as well as between 2760 and 3400 ms (*p* < .001). When we compared “the” and “*el*,” two significant time clusters emerged. Participants looked more at the target for “*el*” than “the” between 1560 and 1780 ms (*p* = .03), but then looked more at the target for “the” than “*el*” between 2100 and 2380 ms (*p* = .015). When we compared “the” and “*la*,” only one significant cluster emerged. Participants looked more at the target for “*la*” than “the” between 1780 and 2500 ms (*p* < .001). Only one of our clusters occurred during the presentation of the noun (between 1200 and 1800 ms), which was for the “*el*”-“the” comparison. The rest of our significant clusters occurred after the presentation of the noun (after 1800 ms).

When we compared English and Spanish nouns in the context of “*el*,” we observed a significant time cluster between 1980 and 2140 ms (*p* = .04). Participants look more at the target when a Spanish noun is presented (e.g., “*el perro*”) than when an English noun is presented (e.g., “*el* dog”). This suggests that participants are sensitive to switches around “*el.*” By contrast, comparing English and Spanish nouns in the context of “*la*” as well as “the” revealed no significant time clusters. This suggests that participants readily processed both single-language (e.g., “*la tortuga*,” “the turtle”) and code-switched phrases (e.g., “*la* turtle,” “the *tortuga*”) containing “*la*” and “the.”

## 4. Discussion

We all use our communicative contexts and grammatical knowledge to facilitate online processing. However, bilinguals experience greater variety in their input that seems to elicit differences in their processing behavior when compared to monolinguals. Some of the relevant literature with Spanish–English bilinguals has largely focused on the variable use of Spanish determiners “*el*” and “*la*” without considering the English determiner “the” due to community norms that largely use Spanish determiners in mixed noun phrases (however, see Blokzijl et al., 2017 for an example of a community where English determiners are preferred). The current study measured and analyzed eye-gaze data as Spanish–English bilingual adults engaged in a visual world to investigate how Spanish–English bilinguals use their grammatical knowledge and linguistic experiences to facilitate processing in both single- and mixed-language contexts. We manipulated what they heard (“find the animal”) to test three hypotheses: (i) whether switching languages impedes processing, (ii) whether grammatical gender facilitates processing and (iii) whether distributional differences between all three determiners elicit processing differences. We also incorporate cluster permutation analyses to test whether processing effects occur during prediction or integration. Based on our results, we argue that Spanish–English bilinguals are sensitive to the distributional norms of all three determiners across both single- and mixed-language contexts. While we do not report the distributional norms of all three determiners as used by the Spanish–English bilingual population in the greater NYC area (reflective of our sample), we report eye-gaze patterns that correspond to observed norms in other U.S.-based Spanish–English bilingual communities.

First, our ANOVA results do not support the proposal that language switching impedes processing. If switching languages is generally costly, we would have expected fewer looks to target for the switch trials (W1, W2, 2X). We would have also likely observed a difference between trials with only one switch (W1, W2) compared to trials with two switches (2X) if switching costs are additive over time. Our ANOVA results did not show a main effect of switch site, but instead an interaction between determiner and switch site. This interaction seems to be driven by “*el*” specifically. Participants looked less at the target when the language of the noun switched (e.g., “*el* dog”) than when the language of the noun stayed the same (e.g., “*el perro*”).

Second, neither the ANOVA results nor the cluster permutation results support the idea that grammatical gender facilitates prediction among our Spanish–English bilinguals. If grammatical gender did facilitate prediction, “*el*” and “*la*” would have elicited greater looks to target than “the.” While we did observe a main effect of determiner from our ANOVA, “*el*” elicited the lowest proportion of looks to target across participants. The cluster permutation results revealed an earlier effect (between 1200 and 1800 ms) for “*el*” that “the,” but not for “*la*” versus “the.” Assuming prediction effects would appear during presentation of the noun, we would have expected an earlier effect for both “*el*” and “*la*” when compared to “the.” However, our results also revealed differences for “*el*” in non-switched (e.g., “*el perro*”) versus switched (e.g., “*el* dog”) contexts. Grammatical gender may override phonological competition between nouns when alternatives are licit (Dahan et al., 2000) but not when the phonological forms come from different languages.

Finally, our ANOVA and cluster permutation results revealed a complex pattern of online processing between all three determiners. Our ANOVA results revealed a main effect of determiner over the entire trial window (0–3500 ms), even though the determiner does not appear until 800 ms after the onset of “find.” Cluster permutation tests were performed to see if differences would emerge after participants received the determiner. Aligned with prior work, participants exhibited greater looks for “*la*” than “*el*” as well as “the.” However, we observed differences for “*el*” when compared with “the” that were surprising: participants first looked more at the target (1560–1780 ms) but then later looked less at the target (2100–2380 ms) for “*el*” than “the.” Our results seem to correspond to distributional differences observed in the literature, with the idea that more restricted cues facilitate processing: “*la*” generally appears before Spanish feminine nouns and is therefore most restrictive in our visual world; “the” generally appears before English nouns; and “*el*” generally appears before Spanish masculine nouns but can also appear before English nouns and is therefore the least restrictive in our visual world. Taken together, our results suggest that bilingual processing differences of noun phrases reflect speakers’ daily experience with determiner-noun distributions across single-language and code-switched contexts.

### 4.1. Reimagining prediction and integration from the bilingual perspective

We standardly treat “*el*” as the Spanish masculine determiner and “*la*” as the Spanish feminine determiner because they typically appear in complementary distribution: both “*el*” and “*la*” appear in similar syntactic environments (before a noun) but co-occur with different types of nouns (masculine [masc], feminine [fem]). Spanish monolinguals seem to use both “*el*” and “*la*” as informative cues because of this distributional pattern (Lew-Williams & Fernald, 2007). However, Spanish monolinguals and Spanish–English bilinguals differ by the availability and productivity of English nouns (which are not subcategorized for gender) as well as “the.” What this could mean for the Spanish–English bilingual is a different function of gender during online processing compared to the Spanish monolingual.

In the context of our visual world paradigm, adopting the standard treatment of Spanish grammatical gender for our Spanish–English bilinguals would predict that the Spanish determiners facilitate processing through pre-activation of plausible Spanish nouns. Our auditory stimuli only presented the Spanish determiners with English nouns that were translational equivalents of the gender-matched Spanish nouns; however, our task involves four areas of interest as opposed to Valdés Kroff et al.’s (2017) study, who use two. Compared to “the,” “*el*” would activate items that refer to the dog (“dog,” “*perro*”) and horse (“horse,” “*caballo*”) and “*la*” would activate items that refer to the chicken (“chicken,” “*gallina*”) and turtle (“turtle,” “*tortuga*”). We observed a facilitatory effect for “*la*” compared to “the” between 1780 and 2500 ms, after presentation of the noun, but not for “*el.*” Instead, we observed an earlier facilitatory effect (1560–1780 ms) and a later costly effect (2100–2380 ms) for “*el*” compared to “the.” We also observed earlier looks to the target when “*el*” is followed by a Spanish noun than an English noun. Despite “*el*” only being viable with the pictures of dog and horse throughout the experiment, participants exhibited an interesting processing pattern for “*el*” that differed from “*la*” and “the.”

One explanation for the differences observed with “*el*” is what our Spanish–English bilinguals, who are generally English-dominant, must resolve during prediction versus integration.^4^ We argue that bilinguals use frequency distributions of phonological forms (e.g., “*el perro*” co-occurs more frequently than “*el* dog”) to predict grammatically licit input when phonological forms are in competition. There has been considerable work to suggest that we use higher-level, abstract representations to predict perceptual inputs, such as phonological forms (Kuperberg & Jaeger, 2015). Our participants are trained to restrict their lexical array of nouns to “dog,” “*perro*,” “horse,” “*caballo*,” “chicken,” “*gallina*,” “turtle” and “*tortuga.*” Recall that, in the Bangor Miami corpus of Spanish–English bilingual speech, 92% of mixed noun phrases combined “*el*” with an English noun compared to the 5% that used “the” with a Spanish noun and 3% that used “*la*” with an English noun (Valdés Kroff, 2012). While the Bangor Miami corpus represents Spanish–English bilinguals from the Miami area, our participants all reside in the greater NYC area. We assume that the gender-assignment pattern observed from the Miami corpus (defaulting to “*el*” for mixed noun phrases) best corresponds to the processing behaviors we observe. Both “*el*” and “the” can co-occur with all four English nouns (as “*el*” has been reported to co-occur with English nouns including Spanish feminine translational equivalents (Beatty-Martínez & Dussias, 2019); but, “*el*” would rank representations with the Spanish masculine nouns “*perro*” and “*caballo*” higher because of its relative frequency of co-occurrence with Spanish masculine nouns. By contrast, “*la*” does not compete with “the” given how “*la*” does not typically co-occur with English nouns.

Having a prediction strategy for phonological forms will, in turn, affect integration through lexical activation. Again, our participants are in a highly restricted context where two phonological forms (e.g., “dog” and “*perro*”) map to a single conceptual representation (dog). “*La*” is the most restrictive in its use, largely co-occurring with Spanish feminine nouns, which would activate the conceptual representations for chicken and turtle. Hearing either the English or Spanish forms for those conceptual representations would be readily integrated based on the evidence showing that the activation of conceptual representations allows for cross-linguistic priming (Francis, 2005) and an assumption that our task would make commitment to a conceptual representation sufficient (albeit ignoring grammaticality or well-formedness) (Karimi & Ferreira, 2016). We can then account for “*la*” eliciting greater looks to target upon hearing the noun than “*el*” and “the” based on how selective the determiner is for activating conceptual representations. However, “*el*” activates all four conceptual representations (because it can also co-occur with English nouns) even though it favors representations predicting the Spanish masculine nouns (given how mixed-language noun phrases occur relatively infrequently). What seems like an asymmetrical switch cost for “*el*” (versus “*la*” or “the”) can then be accounted for as a conflict during integration due to phonological surprisal.

### 4.2. Consequences for bilingual development

Our results support a distributional account for how variability in one’s input affects online processing. Spanish–English bilingual corpora show that “*el*” and “the” both co-occur with English nouns. If these corpora are relatively accurate reflections of what Spanish–English bilinguals receive in their input during development, it is unlikely that the subcategorization of Spanish determiners “*el*” and “*la*” is comparable between Spanish monolinguals and Spanish–English bilinguals. Balam et al. (2021) examine the gender assignment patterns among Spanish–English bilingual children from Miami, Florida, comparable to the adult participants in the Bangor Miami corpus, and found comparable patterns of using “*el*” in mixed noun phrases. One might then predict that the subcategorization of “*el*” that is learned in the Miami community reflects a default use rather than a masculine feature as described for Spanish monolinguals. Our participants from the NYC area, who were exposed to both Spanish and English from early childhood but experienced greater exposure to English in their daily lives, may have regularized a unique default role for “*el*” given its co-occurrence with English nouns and Spanish nouns. However, we do not have information about the nature of our participants’ input and whether this is truly comparable to the Spanish–English bilinguals represented in the Bangor Miami corpus. Future work examining the use of Spanish and English across Spanish–English bilingual communities in the NYC area is needed to corroborate or refute this conclusion.

Furthermore, just as our results may reflect the specific linguistic input experiences of our NYC Spanish–English sample population, and generalizability is an open question, it is also possible that our results are affected by the specific lexical items that we tested. The *el/la* differences we observed may have been affected by the lexical items. A possible factor contributing to the *el/la* differences is that *la* may have been most strongly associated with a single item (*gallina*, “hen”) perhaps due to competition with *pollo* (“chicken”) as a label for the animal and/or the semantic gender associated with hen. This by-lexeme effect would be consistent with our overall conclusion that distributional input patterns affect bilinguals’ predictions and integrations in online processing.

We have learned from studies using artificial languages that distributional information helps shape how lexical items are represented and organized. For example, Reeder et al. (2013) manipulated the degree of overlap between nonce words to see if learners would develop lexical categories implicitly without any semantic or conceptual information. The authors designed an artificial language that contained five lexical categories organized into a canonical word order (QAXBR). Participants who heard sequences with category X words appearing with all of the available category A and category B words (i.e., creating high degree of overlap across contexts) generalized categorical properties; and participants who heard sequences with category X words appearing with only some of the available category A and category B words (i.e., creating low degree of overlap across contexts) did not generalize categorical properties. Similarly, subcategorization properties were generalizable when participants heard sequences with a high degree of overlap for each subcategory (Reeder et al., 2017). Studies using artificial languages also show that the degree of variability in which items appear in similar contexts also yields different outcomes depending on the age of the learner (Austin et al., 2021; Hudson Kam & Newport, 2005). Child learners tend to regularize items that variably appear, whereas adult learners try to match the rate at which items variably appear. One could then interpret from our results that having “*el*” co-occur with English nouns prevents it from developing a subcategorical property among early Spanish–English bilinguals, such as masc grammatical gender, making “*el*” an unmarked Spanish option as opposed to “*la*,” which one could argue develops fem grammatical gender.

What still needs to be tested is whether what we observe with child and adult learners of artificial languages also occurs with child and adult learners of natural languages. Artificial languages are carefully curated such that they lose some of the complexity that we typically see with natural languages. The studies cited here do not make use of semantic/conceptual information and do not take into account the role of prosody as it interacts with both phonological and syntactic representations. Furthermore, studies using artificial languages to simulate bilingual learning environments have not yet demonstrated how bilingual learners of natural languages can differentially acquire both without introducing unnatural adjustments, such as explicit cues to signal a change in language (Weiss et al., 2019).

However, future work using artificial language learning paradigms as well as natural languages, including our ongoing work extending the presented study with toddlers between 2 and 4 years old, will help us understand how distributional cues are used to form grammatical representations, e.g., grammatical gender. Recall that Byers-Heinlein et al. (2017) observed a general switching effect among French–English bilingual toddlers, whereas Potter et al. (2018) observed an asymmetric switching effect among Spanish–English bilingual toddlers. Spanish-learning children in this age range are actively learning the gender agreement system using various cues, including distributional norms (Mariscal, 2009). The differences in task performance between the French–English and Spanish–English toddlers may reflect community specific norms (Aboh & Parafita Couto, 2024) embedded in the distributional nature of determiners and how they are used in mixed noun phrases, such that heritage learners of a given language in a bilingual context develop bilingual-specific grammatical representations rather than behave in a way that reflects the probabilistic norms of their communities (e.g., Fuchs, 2022). We further acknowledge here that future work will need to include some measurements of the participants’ production patterns (such as an elicitation task) and/or linguistic input (such as developing a corpus of production patterns from the participants’ speech community) as our interpretations are limited by not having a better understanding of the Spanish–English bilingual communities in the greater NYC area.

## 5. Conclusion

The current study builds from prior work to investigate how the variability in bilingual input affects online processing. We assert that bilinguals also exhibit incremental processing, engaging prediction and integration mechanisms to facilitate processing. However, bilinguals do not consistently pattern like monolinguals in their online processing behaviors. We used a visual world paradigm with Spanish–English bilinguals to test three hypotheses that would predict different effects between Spanish determiners “*el*” and “*la*” and the English determiner “the.” Our results do not support the idea that grammatical gender, constructed from a monolingual perspective, is an informative cue or that language switching is costly. Rather, they suggest that bilinguals track distributional information across both single- and mixed-language contexts and, in turn, use that information during online processing.

## Footnotes

1 Because the audio files of individual words were presented separately, they re-create a splicing effect because of the pauses varying between words. We recognize that this likely introduces phonological artifacts as co-articulation is not preserved within a given trial. However, this was done to ensure the onsets of each word were comparable across trials.

2 After receiving guidance from a statistician, we wish to highlight that there is a possibility that each unique “find the animal” sequence across our manipulations could elicit systematic variability across our participants. The repeated measures ANOVA conducted here does not capture that possibility, unfortunately. We accept this limitation to present results that most readily compare with the related studies that we reference in motivating this study.

3 We use the term “language switching” here to refer to switches that could happen in either combinatory (e.g., noun phrase: *el* dog) or non-combinatory (e.g., list of nouns: *caballo* dog) contexts. This is meant to be inclusive of studies that have examined language switching effects in non-combinatory contexts (e.g., Meuter & Allport, 1999).

4 We make this interpretation with the assumption that prediction applies during the presentation of a word and integration applies after the presentation of a word. However, this is a rather arbitrary demarcation of time to delineate these two mechanisms. A reviewer raises the possibility that prediction and integration would not apply until ~200 ms after the onset of auditory presentation to account for initial stages of auditory processing.
